# Antibiotic deprescribing: Spanish general practitioners’ views on a new strategy to reduce inappropriate use of antibiotics in primary care

**DOI:** 10.1080/13814788.2022.2130887

**Published:** 2022-10-31

**Authors:** Carl Llor, Gloria Cordoba, Sandi Michele de Oliveira, Lars Bjerrum, Ana Moragas

**Affiliations:** a Department of Public Health, General Practice, University of Southern Denmark, Odense, Denmark; b Health Centre Via Roma, University Institute in Primary Care Research Jordi Gol i Gurina, CIBER de Enfermedades Infecciosas, Instituto de Salud Carlos III, Barcelona, Spain; c International Centre for Antimicrobial Resistance Solutions (ICARS), Denmark; d Section and Research Unit of General Practice, Department of Public Health, University of Copenhagen, Denmark; e Jaume I Health Centre, University Rovira i Virgili, Tarragona, Spain

**Keywords:** Antibacterial agents, deprescribing, surveys and questionnaires, antibiotic stewardship, primary health care

## Abstract

**Background:**

A doctor may recommend that a patient stop an antibiotic course before its scheduled completion time if further treatment may cause more harm than benefit.

**Objectives:**

This study explores general practitioners’ (GP) opinions about the use of antibiotic deprescribing (AD) in general practice.

**Methods:**

A cross-sectional, questionnaire-based study answered from February to March 2022. GPs (*n* = 6,083) affiliated with the largest Spanish scientific society of primary care were invited to participate. The survey included two statements related to use and fourteen views about AD rated by GPs using a 5-item Likert scale.

**Results:**

Eleven hundred and seven doctors completed the surveys (18.2%), of whom 92.5% (95% confidence interval [CI] 90.8–94%) reported having used the AD strategy in their practice at least once. GPs felt very confident in using a deprescribing strategy in patients with common cold and influenza (97.6% and 93.5%, respectively) but less with acute bronchitis (45.5%); 12.1% (95% CI, 10.2–14.2%) considered this practice harmful to patients. Respondents reported using AD more frequently when they initiated the antibiotic course (96.8%; 95% CI, 95.5–97.7) than when the treatment was initiated by another doctor (52.3%; 95% CI, 49.3–55.3%). However, doctors aged >60 years were more prone to use AD compared with younger colleagues (64.5% vs. 50%; *p* < 0.005).

**Conclusion:**

The GPs in this study employ the strategy of AD. Nonetheless, essential differences lie in their views of the way the strategy is used. Further studies are warranted to explore the beliefs behind these perceptions and promote wider use of AD by GPs.

KEY MESSAGES

- Antibiotic deprescribing is used frequently by GPs in Spain.
- GPs do not perceive deprescribing antibiotics as a strategy that can harm patients.
- GPs are more likely to deprescribe an antibiotic course initiated by the patient than one prescribed by a physician.

## Introduction

Dogmas are taken for granted until new evidence poses questions about their validity. A clear example in medicine is the long-standing belief that a course of antibiotics should always last for a defined number of days, irrespective disease’s course and the potential relief of symptoms after fewer days. For several years, patients have always been instructed to complete an antibiotic course once initiated. This was considered appropriate for reducing the development of antimicrobial resistance (AMR) [1], a dogma that has been thoroughly discredited. There is robust evidence of a correlation between consumption of antibiotics and AMR at both the community and individual levels; long-time exposure to antibiotics leads to more AMR than short-time exposure [2,3]. In addition, AMR is more likely if antibiotics use is recent [4]. Good evidence indicates that a reduction of the overall antibiotic consumption leads to a reduced risk of AMR [5,6]. For most infections in primary care, shorter antibiotic treatments are equally effective and are less likely to lead to AMR compared to standard courses, even in life-threatening infections, such as pneumonia [7].

Different strategies have been used to reduce inappropriate consumption of antibiotics in primary care. Most interventions have targeted prescribers and focused on initiatives to reduce the initiation of inappropriate antibiotic treatments. However, few initiatives have been taken to terminate inappropriate treatments already initiated. Most antibiotic prescriptions for respiratory tract infections (RTI) in primary care are inappropriate and fulfilling a course of inappropriate antibiotics may cause more harm than benefit.

Deprescribing is often used in polypharmacy and describes the withdrawal process of an inappropriate medication to improve the overall outcome because continued treatment may cause more harm than benefit [8]. We introduce the concept of antibiotic deprescribing (AD) to characterise the decision to terminate an antibiotic treatment if the doctor assumes that fulfilling the therapy may cause more harm than benefit.

We recently carried out a randomised clinical trial with patients with uncomplicated RTIs who had already taken a dose of an antibiotic course [9]. Among the selection criteria for general practitioners (GP) participating in this trial was the indication that they were comfortable with stopping inadequate antibiotic courses, although only 10 of the 31 were confident in doing so. We found that the mean duration of severe symptoms was similar in the group of patients who discontinued antibiotic therapy and among those who completed the treatment [10]. There is a lack of knowledge about GPs’ views on using AD during daily practice. This study has aimed to help fill this gap by exploring GP use and views about AD.

## Methods

### Design

We conducted a cross-sectional survey among a sample of the Spanish GPs from February to March 2022, using a survey distributed by email. We followed the STROBE guidelines for reporting observational cross-sectional studies [11]. We framed this study within the KAP (Knowledge Attitudes Practices) framework [12].

### Study population

A panel of GPs designed the questionnaires. The survey (Supplementary Material Appendix 1) included several domains of two questions regarding AD use, and fourteen questions about views on AD, including the clinical conditions for which respondents feel confident in deprescribing an antibiotic when no longer necessary and the extent of confidence in stopping an antibiotic course initiated by themselves or other prescribers. Three sociodemographic data and information about their workplace were also collected.

The survey was sent a single time, giving GPs one chance to respond to the questionnaire. Spain’s largest family medicine society is the *Sociedad Española de Medicina de Familia y Comunitaria* (SemFYC; www.semfyc.es, with 20,984 members, a mean age of 37.8 years and 68.6% of female doctors. The inquiry was sent to all the members who previously had consented to receive surveys in their private email box (6,083 GPs).

### Statistical methods

Descriptive and bivariate analyses were carried out. The responses ‘strongly disagree’ and ‘disagree’ were aggregated for simplicity, as were the ‘strongly agree’ and ‘agree’ responses. A chi-square test was used to compare the agreement results on several statements and the diagnoses for which GPs claimed to use AD with age and gender. Data were analysed using SPSS (version 26.0).

### Ethics

This study was granted an exemption from the Ethical Committee Board Jordi Gol i Gurina (Barcelona, Spain), as ethical approval for questionnaire-based surveys is not required in Spain. All the GPs who received this survey had previously consented to receive surveys.

## Results

A total of 2,929 out of 6,083 contacts read or opened the email sent about the study and 1,107 (18.2%) responded and returned the questionnaire. More female than male doctors answered the questionnaire (68%). Most respondents (58%) stated they work as GPs in urban centres. A total of 1,024 (92.3%; 95% confidence interval [CI] 90.8–94%) respondents stated having recommended that patients cease an antibiotic course that they deemed inappropriate (Table 1). A total of 831 GPs (75% of all doctors surveyed) declared that they saw patients whose antibiotic courses were initiated unnecessarily at least once per month.

**Table 1.** Baseline characteristics of participating GPs (*N* = 1,107).

Characteristics	*n* (%)
Gender	
Men	336 (30.4)
Women	753 (68.0)
Age	
<40 years	358 (32.3)
40–60 years	591 (53.4)
>60 years	152 (13.7)
Workplace*	
Doctor in an urban centre	642 (58.0)
Doctor in a rural centre	291 (26.3)
Family medicine trainees	196 (9.6)
Others	68 (6.2)

*Some respondents could have been working in different settings.

**Figure 1.**
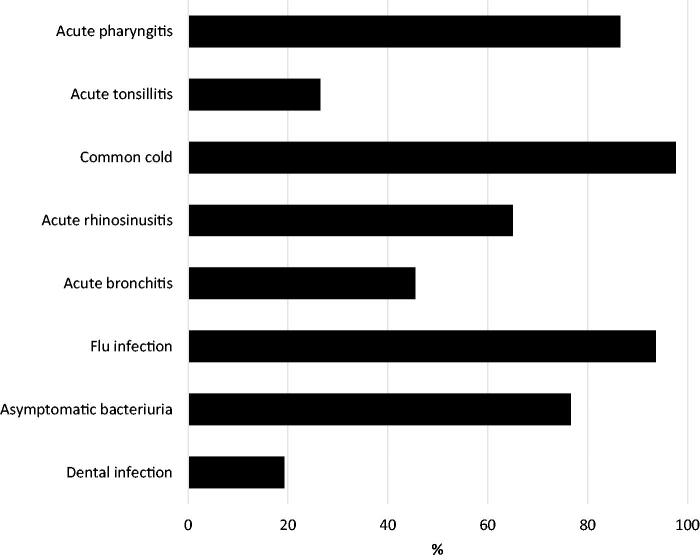
Percentage of GPs willing to deprescribe antibiotic courses stratified by diagnosis (*n* = 1,107).

The clinical conditions for which GPs felt comfortable deprescribing are detailed in Figure 1. These conditions included presumed viral infections, such as the common cold (97.6%; 95% CI, 96.4–98.3%) and influenza infection (93.5%; 95% CI, 92–95%). In contrast, fewer than half of respondents reported feeling confident in stopping an antibiotic course already initiated in patients with suspected tonsillitis (26.5%; 95% CI, 23.9–29.2%), dental infection (19.3%; 95% CI, 17–21.8%) or acute bronchitis (45.5%; 95% CI, 42.6–48.5%).

Regarding attitudes towards AD, only a minority of GPs (12.1%; 95% CI, 10.2–14.2%) considered the practice harmful to patients; the majority view was that AD would not generate an increase in complications and hospital admissions. Thus, the majority (93%; 95% CI, 91.3–94.4%) agreed that this strategy should be included in clinical guidelines. In fact, using the AD strategy received greater favour than the practice of delayed antibiotic prescribing (Table 2).

**Table 2.** Distribution of agreement regarding the practice of antibiotic deprescribing (*N* = 1,107).

	Agree, *n* (%)	Indifferent, *n* (%)	Disagree, *n* (%)
I think that antibiotic deprescribing may generate an increase in complications and hospital admissions.	134 (12.1)	27 (2.4)	946 (85.5)
This strategy should be linked to reconsultation in case of worsening conditions.	1,035 (93.5)	33 (3.0)	39 (3.5)
This strategy should be included in clinical guidelines	1,030 (93.0)	57 (5.2)	20 (1.8)
I recommend this strategy, especially when the patient wishes not to take the antibiotic course.	422 (38.2)	296 (26.7)	389 (34.1)
I prefer using delayed antibiotic prescribing rather than deprescribing an antibiotic when I consider the antibiotic is not needed.	345 (31.2)	244 (22.0)	518 (46.8)
Withdrawing the antibiotic course prescribed by another colleague is unethical.	165 (14.9)	203 (18.3)	739 (66.8)
Deprescribing makes sense in cases of chronic conditions, not in acute conditions.	62 (5.6)	68 (6.1)	977 (88.3)
There is a lack of studies showing that antibiotic deprescribing is safe.	130 (11.7)	335 (30.3)	642 (58.0)
I would only be comfortable in deprescribing if the patient has already taken the medication for at least five days.	133 (12.0)	164 (14.8)	810 (73.2)
I agree to deprescribe if this shows to reduce antimicrobial resistance.	487 (44.0)	192 (17.3)	428 (38.7)
I am comfortable in deprescribing when I believe that in this instance a reasonable conclusion can be made that this strategy will reduce the chance of AMR.	971 (87.7)	62 (5.6)	74 (6.7)
I would act differently during a phone consultation compared to a face-to-face visit.	404 (36.5)	206 (18.6)	497 (44.9)

**Figure 2.**
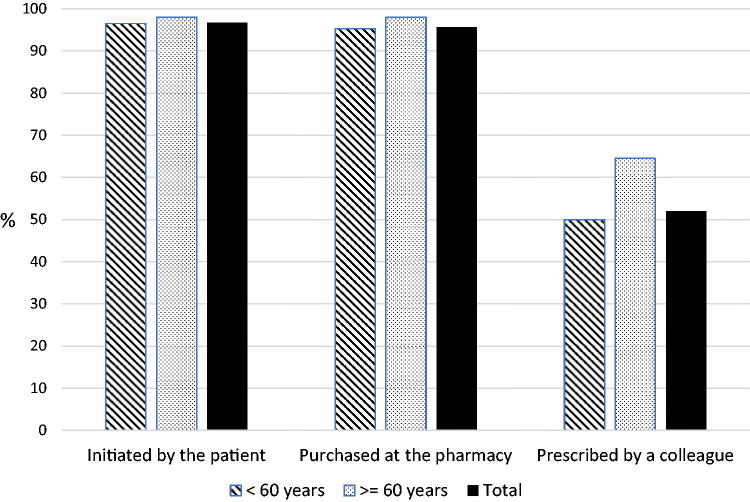
Percentage of GPs willing to deprescribe an antibiotic when deemed no longer necessary depends on how this course was initiated (*n* = 1,107).

Nearly 97% of the respondents endorsed the AD strategy if they deemed that antibiotic treatment was no longer necessary. Respondents recommended AD equally to patients who had initiated treatment themselves and to patients that purchased the antibiotic from a pharmacy without a prescription (96.8%; 95% CI, 95.5–97.7; and 95.7%; 95% CI, 94.3–96.8%). However, only 52.3% (95% CI, 49.3–55.3%) of the respondents agreed to deprescribe an antibiotic regimen, despite deeming it no longer necessary, in instances when another doctor prescribed the antibiotic course. As shown in Figure 2, this percentage was significantly higher among doctors aged more than 60 years than younger colleagues (64.5% vs. 50%; *p* < 0.005). Notwithstanding this, only 14.9% (95% CI, 12.9–17.1%) of the respondents considered it unethical to stop an antibiotic course prescribed by a colleague (Table 2), with this percentage being more significant among doctors younger than 60 (16.4% vs. 5.9%; *p* < 0.001).

## Discussion

### Main findings

In our study, more than 90% of the doctors reported having used the AD strategy at least once in their practice. In addition, 75% of the respondents declared that at least once a month, they see patients whose antibiotic courses are initiated unnecessarily. This strategy is generally performed in viral infections such as common cold and influenza but not in others, such as acute bronchitis. Only 45% of GPs agreed to perform AD in patients with acute bronchitis who had already commenced an antibiotic course. Most GPs felt comfortable ceasing an antibiotic course initiated from leftovers in the household or obtained at the pharmacy without a prescription. However, only about half of GPs considered AD appropriate if a colleague had started the antibiotic course.

### Strengths and limitations

To our knowledge, this is the first time this topic has been studied in primary care. Its novelty and the large sample of responses are the major strengths of this study. Conducting online surveys with health professionals has several advantages in comparison to mailed questionnaires, as it is easier to implement large-scale surveys at a low cost [13]. Another advantage of electronic questionnaires is that data are automatically transferred to a database, eliminating the need to enter the data manually and avoiding potential data entry errors. However, external validity is a primary concern, specifically how to obtain a representative sample and good response rate [14]. This response bias, with respondents possibly having been more interested in this topic than non-respondents, must be acknowledged, as in our study only 18.2% of all responses were received from those who opened or read their personalised email message. Respondents were a bit older compared to the mean age of society members with a similar percentage of female doctors. In addition, one cannot assume that registered members of a specific scientific society will necessarily reflect the whole group of these health professionals. The results of this study can only be extrapolated to the reality of high antibiotic consumption areas. The antibiotic prescribing rates vary substantially across countries, and the results obtained in our study could have been different in countries with lower antibiotic use. In addition, not all the doctors in any given country feel comfortable when withholding antibiotic therapy for self-limiting RTIs, let alone deprescribing antibiotic courses.

Another weakness of these types of studies is the gap between opinion and actual practice. As in other opinion studies, the results obtained do not accurately reflect actual primary care practices. Rather, they correspond to hypothetical scenarios in which GPs were asked to give their opinion about each situation. Despite these weaknesses, this manuscript provides valuable insights into how GPs perceive the use of AD as a treatment strategy and serves as a point of departure for future qualitative studies.

### Comparison with existing literature

The term ‘deprescribing’ is mainly linked to chronic diseases, as it is increasingly recognised that many adults and older people take a large number of medications, often including unnecessary or potentially inappropriate ones [15]. However, deprescribing can also be performed in acute conditions. The best way to minimise unnecessary medication use is to be judicious in prescribing medications in the first place. However, this decision might have been made by a colleague, a pharmacist, a private doctor, or even by the patients (self-prescribing), who come to our practice for reassurance. Once the therapeutic decision has been made and the patient has already taken one or a few doses of an antibiotic course, healthcare professionals must make their best judgement on the appropriateness of continuing this medication after thoroughly considering the patient’s history and clinical examination. If we assess that the antibiotic course will result in more harm than benefit, we should pursue the cessation of the therapy. We need to overcome the usual prescribing inertia that is so common in some chronic diseases but also, unfortunately, is so widespread with antimicrobial therapy [16].

When it comes to antimicrobials, this inertia can even be more harmful than with chronic medications, as the emergence and spread of AMR can make the treatment of infectious diseases challenging in the future. Even short courses of antibiotics can have long‐term effects, causing the persistence of resistant organisms lasting years and altering the normal gut microbiome and might be detrimental [17]. Beyond recommending shorter courses of antibiotics, discontinuing antibacterial drugs as soon as a bacterial infection has been reasonably ruled out also helps reduce the development of common side-effects such as diarrhoea and rash [18].

Many GPs would consider that discontinuing antibiotics in these circumstances should be the standard of care. However, the decision to discontinue an antibiotic recently started is not easy, particularly if initiated by a colleague. In fact, as suggested by qualitative research, GPs do not feel comfortable discontinuing treatments in general, and guidelines are not as authoritative for discontinuation as they are for starting drugs [19]. Doctors consider it their priority to be responsive to the needs of the individual patient, viewing AMR as a public health issue. Some clinicians do not feel it is worth jeopardising their relationship with a patient over what they consider a relatively minor matter, prescribing antibiotics [20], or over concerns of AMR [21].

Another factor to highlight is that many GPs do not wish to contradict the decision made by other doctors because it might be viewed as unfair [22]. This phenomenon, referred to as ‘prescribing etiquette’, is a crucial determinant of behaviour, with prescribing decisions influenced not only by clinical goals but also by cultural determinants [23]. In our study, only half of the GPs agreed to deprescribe when a colleague initiated the antibiotic, mainly because they do not wish to offend a colleague who decided to start an antibiotic course. In this study, senior doctors tend to set aside the so-called ‘prescribing etiquette’. This might indicate that hierarchy and experience mitigate the influence of this etiquette. Doctors working at emergency departments who might have prescribed an unnecessary antibiotic tend to be younger – many trainees and junior doctors are hired in this setting, and this could explain why senior doctors are more confident in stopping unnecessary antibiotic treatments than their younger counterparts.

### Implications for practice

The inappropriate use of antibiotics for uncomplicated RTIs is very high [24–26]. Many strategies aimed at reducing unnecessary antibiotic use have been suggested, including guidelines, education, audits and feedback, as well as delayed prescriptions. However, early discontinuation of antibiotics (AD), when the prescription is deemed unnecessary after a clinical re-evaluation, should become the norm [27]. However, discontinuing an unnecessary antibiotic course is seldom carried out in routine clinical practice because most physicians are concerned about hypothetical negative consequences of discontinuation. Fear of complications is one of the main reasons doctors feel reluctant to stop an antibiotic course despite knowing that this is no longer needed [28]. Notwithstanding this, only 12% of the respondents considered that the AD practice could lead to more complications and hospital admissions.

The results of this questionnaire-based study show that healthcare professionals are reluctant to question the prescribing habits of their peers. In the case of antimicrobials, prescribing etiquette and its role as a key determinant of prescribing behaviours is highly significant, recognising the autonomous decision-making process of prescribing. These findings have clear implications for quality improvement interventions in antimicrobial prescribing. Therefore, understanding and addressing the determinants of antimicrobial prescribing behaviours hold the key to successful quality improvement intervention. Further qualitative research is needed to understand how to tackle ‘prescribing etiquette’ as part of the cultural and organisational factors that shape the GPs’ decision to prescribe.

## Conclusion

This paper shows that AD for acute conditions is used by GPs and highlights an array of beliefs about AD. Despite being deemed as a novel strategy to reduce antibiotic consumption, this practice is often used in our consultations, but probably not as much as could be warranted. We, therefore, have an ethical demand to stop this course when the clinician judges that it is no longer needed.

## Supplementary Material

Appendix 1
