# Supplementary material for: Antibiotic deprescribing: Spanish general practitioners’ views on a new strategy to reduce inappropriate use of antibiotics in primary care
Source: Eur J Gen Pract. 2022 Oct 31;28(1):217–23. doi: 10.1080/13814788.2022.2130887 (PMC9629099; doi:10.1080/13814788.2022.2130887)
Supplement: Appendix 1 [file IGEN_A_2130887_SM4928.docx]

**APPENDIX 1. Original survey sent to members of the Spanish Society of Family Medicine (SemFYC) who consented to receive surveys in their private emails**

La semFYC lleva desde 2011 promoviendo el uso racional de antibióticos y ha trabajado activamente con el objetivo de deprescribir una pauta antibiótica cuando el clínico considera que ésta no es necesaria. El Grupo de Trabajo de la semFYC en Enfermedades Infecciosas ha elaborado esta encuesta dirigida a conocer su uso por parte de los socios y las socias de la semFYC. En este sentido nos gustaría que pudieras participar en ella, que no ocupa más de tres minutos. Te rogamos que contestes con la máxima sinceridad. Gracias.

1. **¿Has quitado alguna vez un tratamiento antibiótico iniciado ya por el paciente porque considerabas que el paciente no lo necesitaba?**

Sí, alguna vez lo he hecho

Nunca lo he hecho

1. **¿Con qué frecuencia te encontrabas antes de la pandemia con pacientes que habían iniciado una pauta antibiótica que tú no habías prescrito?**

Menos de un caso al mes

Uno o dos casos al mes

Un caso a la semana

Más de un caso semanal

1. **Estaría de acuerdo en quitar una pauta antibiótica cuando considero que el paciente no lo necesita**

| Enunciado | Totalmente en desacuerdo | En desacuerdo | Indiferente | De acuerdo | Totalmente de acuerdo |
| --- | --- | --- | --- | --- | --- |
| Si es el paciente quien la inició |  |  |  |  |  |
| Si fue dada en la farmacia sin receta |  |  |  |  |  |
| Si fue prescrita por otro médico en otro centro |  |  |  |  |  |
| Si fue prescrita por un médico privado (p.ej. dentista) |  |  |  |  |  |
| Si el paciente es reacio a tomarla |  |  |  |  |  |
| Si presenta algún evento adverso leve |  |  |  |  |  |

1. **Di si estás de acuerdo con los siguientes enunciados sobre deprescripción antibiótica**

| Enunciado | Totalmente en desacuerdo | En desacuerdo | Indiferente | De acuerdo | Totalmente de acuerdo |
| --- | --- | --- | --- | --- | --- |
| Pienso que esta práctica puede generar un aumento de complicaciones y hospitalizaciones |  |  |  |  |  |
| El uso de esta estrategia debería estar ligada a un plan de reconsulta en caso de empeoramiento |  |  |  |  |  |
| Debería estar recogida en las guías de práctica clínica |  |  |  |  |  |
| La recomendaría si el paciente pide específicamente no tomar antibiótico |  |  |  |  |  |
| En general prefiero usar la prescripción diferida de antibióticos y ser el paciente quien decida |  |  |  |  |  |
| No me parece ético retirar el antibiótico prescrito por otro profesional |  |  |  |  |  |
| La deprescripción solo tiene sentido en patologías crónicas, no en una patología aguda |  |  |  |  |  |
| Faltan aún estudios que evidencien que la deprescripción antibiótica es segura |  |  |  |  |  |
| Solo estaría seguro de hacerlo si el paciente ha tomado la medicación durante al menos 5 días |  |  |  |  |  |
| Estoy de acuerdo de hacerlo solo si esto demuestra reducir resistencias antimicrobianas |  |  |  |  |  |
| Pienso que esta práctica puede ayudar al paciente a que haya más conciencia sobre el problema de las resistencias |  |  |  |  |  |
| Actuaría distinto si es una visita presencial o telefónica |  |  |  |  |  |

**5. ¿Con qué patologías te sentirías más cómodo deprescribir una pauta antibiótica? (marca todas las que consideres adecuadas)**

Faringitis aguda

Amigdalitis aguda

Catarro común

Rinosinusitis aguda

Bronquitis aguda

Gripe

Bacteriuria asintomática

Infección odontológica

En ninguna

**Sexo**

Hombre

Mujer

**Edad**

<40a

40-59a

60a o más

**¿Dónde trabajas?**

Centro de salud urbano

Centro rural

Centro de urgencias

Hospital

MIR

Otro

**Muchas gracias por tu participación. Si quieres comentar algo, escríbelo aquí**.
